# Regulatory T cells in rheumatoid arthritis: functions, development, regulation, and therapeutic potential

**DOI:** 10.1007/s00018-022-04563-0

**Published:** 2022-09-29

**Authors:** Shuaifeng Yan, Konstantin Kotschenreuther, Shuya Deng, David M. Kofler

**Affiliations:** 1 Laboratory of Molecular Immunology, Division of Rheumatology and Clinical Immunology, Department I of Internal Medicine, Faculty of Medicine and University Hospital Cologne, University of Cologne, Kerpenerstr. 62, 50937 Cologne, Germany; 2 Center for Molecular Medicine Cologne (CMMC), University of Cologne, Cologne, Germany; 3 Department of Ophthalmology, University of Cologne, Cologne, Germany; 4 Center for Integrated Oncology, Aachen Bonn Cologne Duesseldorf, Cologne, Germany

**Keywords:** Regulatory T cells, Chimeric antigen receptor, CD4+ T cells, Therapeutic potential, Self-tolerance, Autoimmunity

## Abstract

Rheumatoid arthritis (RA) is an autoimmune disease that mainly affects the joints but also leads to systemic inflammation. Auto-reactivity and dysregulation of self-tolerance are thought to play a vital role in disease onset. In the pathogenesis of autoimmune diseases, disturbed immunosuppressive properties of regulatory T cells contribute to the dysregulation of immune homeostasis. In RA patients, the functions of Treg cells and their frequency are reduced. Therefore, focusing on the re-establishment of self-tolerance by increasing Treg cell frequencies and preventing a loss of function is a promising strategy for the treatment of RA. This approach could be especially beneficial for those patients who do not respond well to current therapies. In this review, we summarize and discuss the current knowledge about the function, differentiation and regulation of Treg cells in RA patients and in animal models of autoimmune arthritis. In addition, we highlight the therapeutic potential as well as the challenges of Treg cell targeting treatment strategies.

## Introduction

Rheumatoid arthritis (RA) is an autoimmune disorder characterized by chronic inflammation in multiple joints, inducing synovitis, cartilage damage and bone erosion. Joint destruction can lead to disability, reduced life quality, reduced life expectancy, and a high burden on the healthcare systems [1, 2]. RA affects about 1% of the population worldwide, occurs at any age and affects women two to three times more often than men [3–5]. Currently, conventional synthetic disease-modifying antirheumatic drugs (DMARDs), targeted synthetic DMARDs, and biological DMARDs are used in clinical practice and can induce remission in many patients. However, in approximately 30% of the patients remission cannot be achieved and RA remains an incurable disease due to the complexity of its pathogenesis [6–9]. Thus, it is necessary to identify new therapeutic targets for the treatment of RA patients, especially for those who do not respond to current therapies.

In the past years, reports about the frequency of Treg cells in the peripheral blood and in the synovial fluid of RA patients have shown contradictory results [10–12]. The differences between the reported results might be due to different approaches used to identify Treg cells. Treg cells seem to be significantly decreased in the peripheral blood at the early stage of RA [13]. Moreover, Treg cells accumulate in the synovial fluid and synovial membrane of inflamed joints of RA patients [14]. The frequency of Treg cells in synovium, peripheral blood and synovial fluid might affect the cell contact-mediated suppressive function of Treg cells in RA [15]. Therefore, both the lack of Treg cells and impaired Treg cell functions contribute to the imbalance between effector T cells and regulatory T cells in RA. Importantly, in humans and mice with Foxp3 deficiency can lead to a proliferative autoimmune disorder, whereas the transfer of Foxp3^+^ Treg cells helps to prevent the development of a fatal lymphoproliferative syndrome with inflammation in many organs caused by the deficiency of foxp3 gene in mice [16]. Lentiviral overexpression of FoxP3 in CD4^+^ T cells from RA patients induces increased levels of CD25 and CTLA-4 and decreased levels of CD127 and TNF-alpha [17]. Considering the important role of Treg cells in immune homeostasis, reestablishment of self-tolerance by Treg cell therapy seems to be a promising approach to reduce autoimmunity in RA patients. In this review, we will discuss the current knowledge about the function, differentiation and regulation of Treg cells in RA and murine arthritis models In addition, we will highlight the therapeutic potential and challenges of Treg cells and the progress in this field.

## Suppressive properties of regulatory T cells

Regulatory T cells are a CD4^+^ T cell subset that is characterized by expression of its master transcription factor Foxp3 (Forkhead box protein 3), high expression of IL-2 receptor (CD25) and low or negative expression of CD127 [18, 19]. In addition, a small subset of Foxp3 negative CD4^+^ T cells which is characterized by TGF-beta1 and IL-10 secretion has similar suppressive abilities as Foxp3^+^ Treg cells [20–22]. This subset is called type 1 regulatory T cells (Tr1 cells). However, the surface markers and transcription factors of this subset have not been fully identified [23]. To date, CD4^+^CD25^high^CD127^low^Foxp3^+^ cells remain the most studied Treg group. Our review will, therefore, mainly focus on this cell type, which is crucial for the prevention of autoimmunity despite its low frequency in the peripheral blood [24–27].

### Treg cell transcription factors

Foxp3 promotes the differentiation of naïve CD4^+^ T cells into Treg cells and is the most important transcription factor for the development and function of Treg cells [28]. Scurfy mice which are deficient for Foxp3 die early due to highly activated CD4^+^ T cells and overwhelming proinflammatory cytokine production [29]. Humans with a mutation in the Foxp3 gene can develop the autoimmune inflammatory syndrome IPEX which is characterized by immune dysregulation, polyendocrinopathy and enteropathy [30]. Furthermore, the overexpression of Foxp3 helps to increase the absolute number of Treg cells and CD4^+^CD25^−^Foxp3^−^ T cells transfected with Foxp3 show immune suppressive properties and prevent autoimmunity in a mouse model This evidence indicates that the transcriptional factor Foxp3 is crucial for maintaining the suppressive activity of Treg cells, both in human and mice. However, it seems that Foxp3 is not the only gene required for the maintenance of Treg cell development and function. It has been shown that Helios enhances Treg cell function in cooperation with Foxp3. Helios increases the suppressive function of induced Treg cells and upregulates various Treg cell-related molecules [31]. Moreover, Zheng et al. reported an important role of conserved non-coding DNA sequence (CNS) elements at the Foxp3 locus in the determination of Treg cell frequency, stability and characteristics in mice [32]. Furthermore, CTLA4-Ig and vasoactive intestinal peptide (VIP) are also reported to play a role in the development of Treg cells [33, 34]. However, it remains elusive how these proteins work together to determine Treg cell development and suppressive activity.

### Suppressive mechanisms

The suppressive activity of Treg cells is mediated by different mechanisms, including cytokine production, direct cell–cell contact suppression and the regulation of antigen-presenting cells (APCs), which induce effector T cells apoptosis and immunosuppression [35–39].

#### Cytokines produced by Treg cells

**Fig. 1:**
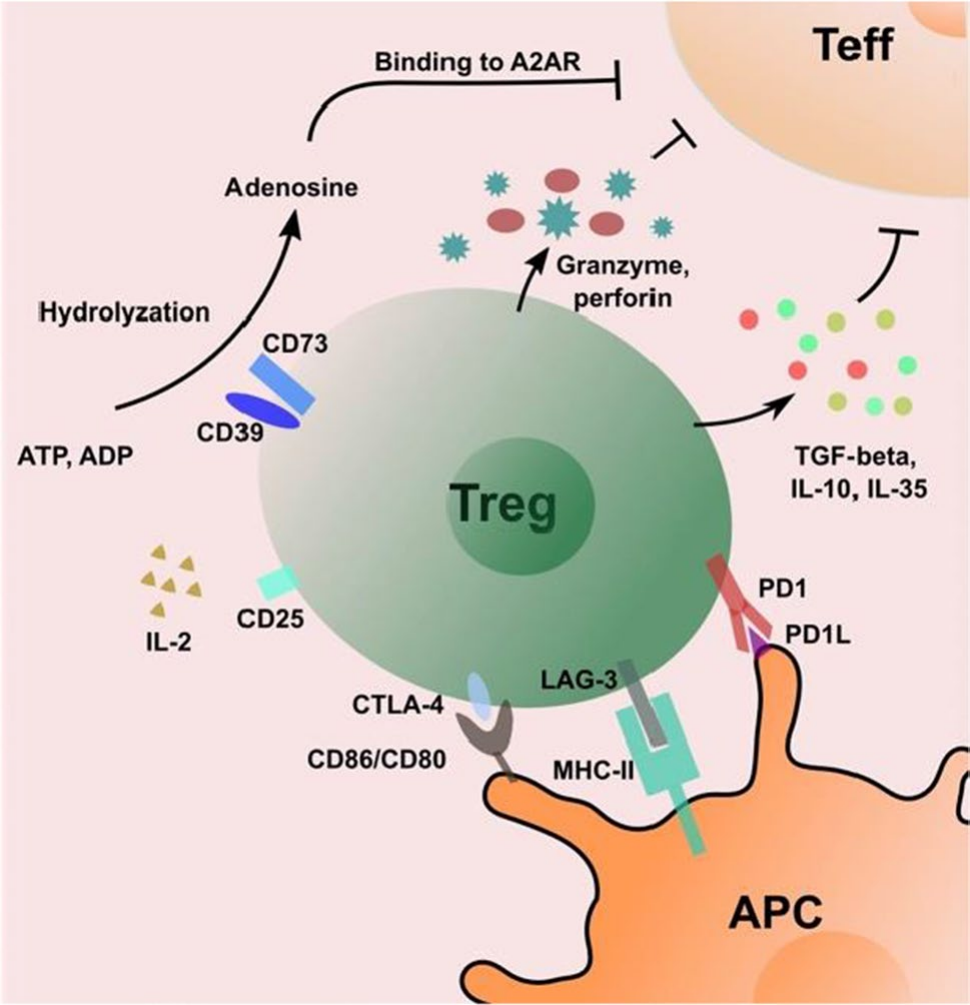
The mechanisms of immunosuppressive function mediated by Treg cells. Treg cells secret the cytokines TGF-beta, IL-10 and IL-35 to directly inhibit the activation and proliferation of effector T cells. CTLA-4, LAG-3 and PD1 on Treg cells mediate the downregulation of APC cell functions, which prevents the activation of naïve T cells and effector T cells. CD25 expressed on Treg cells outcompetes IL-2 which is necessary for T cell in the peripheral to prevent its activation and proliferation. Adenosine produced by the hydrolyzation of CD39 and CD73 from ATP or ADP binds to A2A receptor on effector T cells, thereby inhibiting the proliferation and production of inflammatory cytokines. Treg cells also mediate the cytolysis of effector T cells by granzymes and perforin

Treg cells produce a series of cytokines that contribute to their suppressive function. These cytokines include IL-10, TGF-beta and IL-35 (as shown in Fig. 1) [40–44]. In the DBA/1 mouse line, heterozygous (IL-10^±^) and homozygous (IL-10^−/−^) mice develop worse arthritis compared to wildtype (WT) mice following induction of collagen type II [45]. Similar results have been observed in C57BL/10 mice and IL-10 deficient mice [46]. Anti-IL-10 antibodies inhibit the expansion of Treg cells in mice with collagen-induced arthritis (CIA), whereas human IL-10 gene transduction can ameliorate the symptoms of established experimental autoimmune arthritis [47, 48]. In addition, it has been reported that Tr1 cells characterized by IL-10 production have the suppressive capacity in mice and humans [49]. Moreover, TGF-beta produced by Treg cells promotes their suppressive function, the expression of Foxp3 and immune homeostasis in vivo [50]. Treg cells also consistently express a high level of TGF-beta on their cell surface after stimulation, which contributes to the inhibition of effector T cells activation and proliferation in both, human and mice [51, 52]. TGF-beta1 produced also mediates immunosuppression by restricting the production of immunoglobulins (Ig) by B cell. These inhibitory effects can be disrupted by the treatment with anti-TGF-beta antibodies [52]. In addition, IL-35 is constitutively expressed in murine Treg cells and contributes to the inhibitory function of Treg cells [43]. Finally, Nakano et al. reported that IL-35 induces suppression of peripheral T cells in RA patients [53]. Taken together, various cytokines help Treg cells to exert maximal suppression of activated immune cells.

#### Cell–cell contact-mediated suppression

A two-step mode of cell contact-mediated suppression by Treg cells was proposed by Onishi et al.: First, leukocyte function-associated antigen-1 (LFA-1) dependent formation of Treg cells facilitates the interaction with immature dendritic cells, followed by subsequent upregulation of LFA-1 and cytotoxic T-lymphocyte-associated antigen 4 (CTLA-4) on activated Treg cells. These molecules can interact with antigen-presenting dendritic cells (DC) and lead to reduced expression levels of CD80 and CD86 on DCs, thus preventing the activation and proliferation of antigen-reactive naïve T cells by decreasing the ability of APC both in vivo and in vitro [54]. Like CTLA-4, the transmembrane protein lymphocyte activation gene-3 (LAG-3) binds MHC II on antigen-presenting cells and inhibits DC activation through an ITAM-mediated inhibitory signaling pathway [55]. Furthermore, the CD28 superfamily member PD-1 binds to its ligands, thereby preventing proliferation and IFN-γ production in T cells [56]. Importantly, granzyme B expressing Treg cells have cytotoxic effects on effector T cells [57] and the apoptosis of effector T cells can be induced by Treg cells in a cell–cell contact-dependent manner [58]. In summary, these suppressive pathways work together to induce cell contact-dependent suppressive function of Treg cells (as shown in Fig. 1). Recently, it was reported that murine Treg cells also mediate suppressive functions through CD40L and P-selectin-dependent pathways during cell–cell contact-dependent inhibition [59]. So far, it is not fully understood how this cell contact-dependent suppression is regulated.

#### Regulation of metabolism in target cells

Another mechanism of Treg cell suppression is the metabolic disruption of the targeted cells. Treg cells are characterized by high expression levels of IL-2R (CD25) [60]. This helps Treg cells to bind IL-2 and to prevent IL-2 consumption by effector T cells. IL-2 is crucial for the maintenance of effector T cells activation and proliferation. Lack of IL-2 induces apoptosis of effector T cells [39]. Moreover, Treg cells also mediate immunosuppression by the ectonucleotidases CD39 and CD73. CD39 is constitutively expressed on Treg cells and can hydrolyze ATP and ADP [61]. It also acts together with CD73 to produce adenosine (Fig. 1) [62]. Once binding to adenosine receptor A2A on activated T cells [63], adenosine can induce metabolic disruption through the following mechanisms: cyclic adenosine monophosphate (cAMP) in Treg cells drives the inhibition of T cell proliferation and promotes the synthesis of IL-2 by gap junction formation, which prevents the production of proinflammatory cytokines in effector T cells [64].

## Development of Treg cells

To date, three different phenotypes of Treg cells have been identified based on their origins: natural Treg (nTreg) cells derived from the thymus, peripheral Treg cells from peripheral lymphoid organs and induced Treg (iTreg) cells differentiated in vitro from naïve T cells under Treg skewing conditions [65]. All of them are characterized by the expression of Foxp3 [30].

### Natural Treg cells

In the thymus, nTreg cells are derived from hematopoietic progenitor cells through a two-step mechanism [66] and migrate into the peripheral blood to maintain immune tolerance and to prevent autoimmunity. Early T cell development occurs in the cortex whereas the later phases of maturation occur in the medulla [67]. Immature CD4^+^ single positive T cells get depleted by strong TCR activation, whereas the cells receiving intermediate TCR activation escape from negative depletion and are able to differentiate into Treg cells [68]. First, TCR stimulation and cytokines drive the CD4^+^ single positive T cells to upregulation of IL-2R and TNF receptor superfamily members (GITR, OX40, and TNFR2). Next, the expression of FoxP3 is upregulated by recognition of self-antigen-MHC II complexes presented by thymic antigen-presenting cells, which results in the maturation of Treg cells [69–72]. Natural Treg cells are fairly stable and Foxp3 is stabilized by demethylation of the CNS2 region of the Foxp3 locus that leads to recruitment of various transcription factors including Foxp3 itself [73, 74].

### Peripheral and induced Treg cells

During the development of peripheral Treg cells, naïve CD4^+^ T cells first migrate into the peripheral blood without any TCR activation. Once stimulated by antigens in the peripheral blood, naïve CD4^+^ T cells can differentiate into Foxp3^+^ Treg cells in the presence of both, TGF-beta and IL-2 [75]. Despite their low frequency, peripheral Treg cells can prevent inflammation in barrier tissues [72, 76, 77]. When Treg cells are generated in vitro, they are termed iTreg cells [78]. These cells are also suppressive and can maintain immune homeostasis [79]. Moreover, retinoic acid in the gut is also reported to promote peripheral Treg cells differentiation [78]. Mucosal dendritic cells induce Foxp3^+^ Treg cells by producing TGF-beta and retinoic acid [80, 81]. The microbial metabolites are short-chain fatty acids (SCFAs) and were reported to facilitate peripheral Treg cell development. However, TGF-beta is required for regulatory effects [82]. It has to be emphasized that iTreg cells are not so stable compared to nTreg cells.

### Other suppressive CD4^+^ T cell populations

There is an additional CD4^+^ T cell subset that mediates immunosuppression in vitro and which attracted a lot of attention in the past years. The cells are characterized by secretion of TGF-beta1 and IL-10 without expressing the transcription factor of Foxp3 [20]. This subset is termed type 1 regulatory T cells (Tr1 cells). However, no specific surface biomarker or transcription factors for this subset have been identified so far, although some promising candidates have been reported [23]. The suppressive mechanisms used by Tr1 cells are similar to those of nTreg cells and include the production of immunosuppressive cytokines, cell–cell contact-mediated suppression, cytotoxicity, and metabolic disruption [23]. IL-10 and other cytokines, including IFN-α, IL-6 and IL-27, are required for the generation of Tr1 cells [83–86]. Importantly, Tr1 Treg cells are found to be less suppressive compared to nTreg cells in the early stage of life, because they fail to rescue IPEX patients with a complete lack of Foxp3 [21]. In addition, it has been also reported that Foxp3^+^ CD8^+^ T cells, CD4^−^CD8^−^ cells and gamma/delta T cells also share some suppressive properties, but no evidence has shown that they play an important role in self-tolerance [30, 87].

## Regulation of Treg cells in rheumatoid arthritis

Compared to healthy individuals, significant lower frequencies of Treg cells are found in the peripheral blood of patients with RA at an early stage of disease [88]. Moreover, a negative correlation between CD4^+^CD25^high^CD127^low^ Treg cell numbers and disease activity as assessed by disease activity score 28 (DAS-28) has been reported [89]. Treg cells are also found in the synovial fluid of RA patients, but their suppressive function is impaired [90]. In a study comparing Treg cell numbers in the synovial fluid of RA patients and osteoarthritis patients, the number of Treg cells were increased in the synovium of RA patients but failed to suppress dendritic cells activation and maturation [91]. In addition, Treg cells in the synovial fluid express higher levels of CTLA-4, GITR, OX40, and Foxp3 despite their impaired ability to suppress the proliferative response of effector T cells [12]. Treg cell plasticity has been reported to allow Treg cells to develop into highly inflammatory Th17 cells under physiological and pathogenic conditions. Interestingly, some Treg cells in the peripheral blood of RA patients produce IL-17 while they maintain their suppressive function [92]. However, it remains unknown if IL-17-producing Treg cells contribute to the pathogenesis of RA. Ex-Foxp3 T cells represent another T cell subset that is found in RA patients, Their phenotype is less stable in inflamed joints with high IL-6 concentration and they do not express Foxp3. These ex-FoxP3 T cells can convert into more osteoclastogenic Th17 cells as compared to Th17 cells which have been differentiated from naïve T cells [93]. Furthermore, deficiency of genes encoding for IL-2, IL-2 receptor (CD25) or CTLA-4 are associated with autoimmune diseases, indicating that these Treg associated cytokines and molecules are important for the prevention of autoimmune disorders [94–97]. In the murine model of collagen-induced arthritis (CIA), the suppressive capacity of Treg cells from mice immunized with complete Freud’s adjuvant (CFA) and collagen type II is impaired. Disruption of Treg cells by anti-CD25 antibodies in these mice has been shown to accelerate the onset of joint inflammation and to increase disease severity. Moreover, iTreg cells from CIA mice upregulate FoxP3 following cell contact with dendritic cells (DC). DC-stimulated Treg cells can prevent effector T cell proliferation and Th17 cell differentiation more efficiently than iTreg cells without DC stimulation, leading to less severe arthritis [98]. In summary, various studies indicate that dysregulation and deficiency of Treg cells play an important role in the pathogenesis of RA.

## Therapeutic potential of Treg cells

**Fig. 2:**
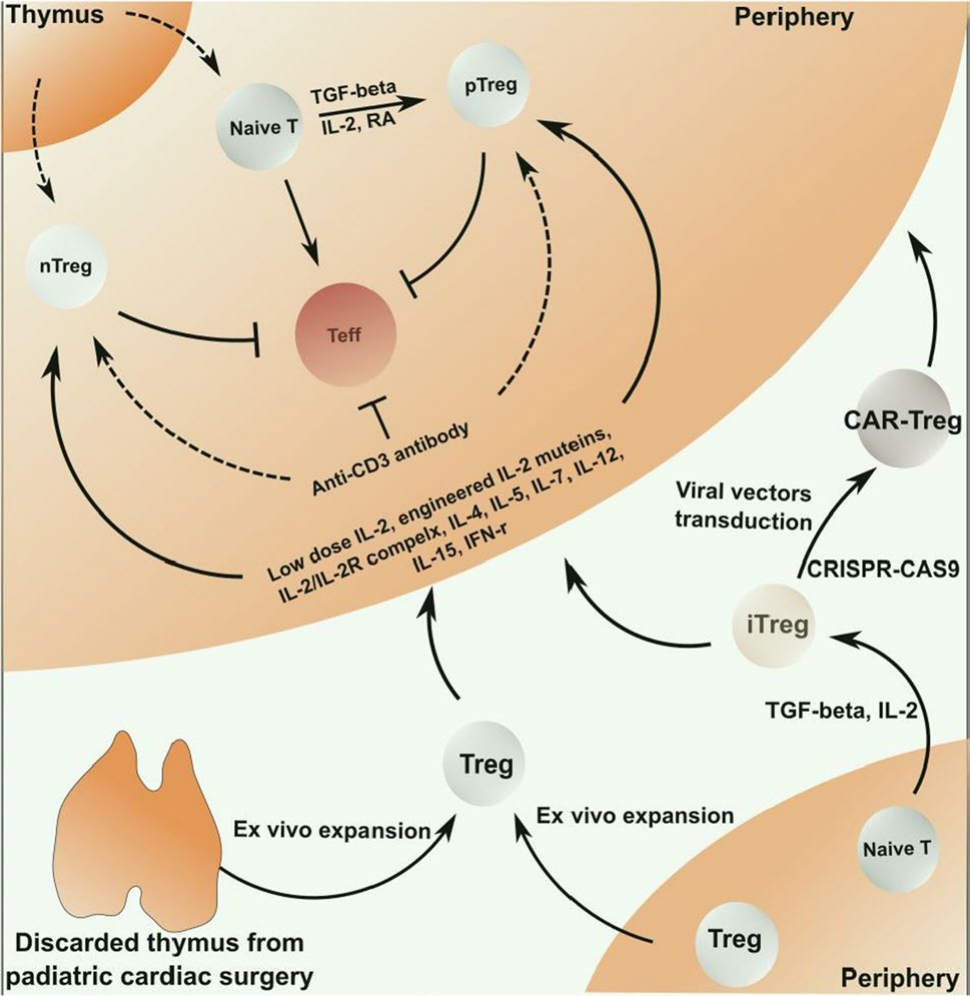
Strategies to increase Treg cells frequencies and the suppressive function in vivo and in vitro. In vivo and in vitro strategies can be used to increase Treg cells number or function with immunomodulatory drugs. Low dose IL-2, engineered IL-2 muteins, the complex of IL-2/IL-2 receptor, IL-4, IL-5, IL-7, IL-12, IL-15, and IFN-γ are shown to have the potential to increase the activity of natural Treg cells or peripheral Treg cells against effector T cells in vivo. In addition, selective depletion of effector T cells by anti-CD3 antibodies can restore Treg cell predominance over effector T cells. Treg cells or naïve T cells isolated from the peripheral blood or the thymus of pediatric cardiac patients can be expanded and genetically modified in vitro for adaptive transfer to increase Treg cell numbers or improve specificity

Treg-based therapies are a promising approach to overcome impaired Treg cell functions and reduced frequencies of Treg cells in RA. The therapeutic potential of Treg cells has been shown in other autoimmune disorders, including experimental autoimmune diabetes and encephalomyelitis [99, 100]. Preclinical research indicates that Treg cell therapies can delay the onset of autoimmune inflammation and reduce graft-versus-host reactions in organ transplantation [101]. Application of type 1 regulatory T cells to symptomatic patients with refractory Crohn’s disease has been proved to be safe and to achieve dose-dependent effects [102]. Moreover, ex vivo expansion of polyclonal Treg cells from patients with early type 1 diabetes resulted in a significant increase in Treg cells numbers, C-peptide levels, prolonged survival of beta-cells, and lower requirement for insulin [103]. In addition, regulatory T-cell therapy proved to be safe in patients who received kidney transplantation, leading to less infectious complications despite an equivalent rejection rate in the first year [104]. Currently, Treg cell therapies are evaluated in several phase I/II clinical trials involving patients with cutaneous lupus, type 1 diabetes, Crohn’s disease, autoimmune hepatitis, and organ transplantation [105]. The results of these trials will help to improve the design of future Treg-based therapies for RA patients. The current strategies to increase Treg cell numbers and their function in vivo and in vitro are shown in Table 1 and in Fig. 2.

**Table 1:** Overview of common in vivo and in vitro Treg-based therapeutic strategies

	Immunomodulatory interventions	Mechanisms	Effects on Treg cells	Model/disease
In vivo	Low-dose interleukin-2 [110, 111, 113]	Inflammation and oxidative stress mediators attenuation, Endogenous immune tolerance restoration	Treg cells expansion, activation, and function activation	HCV-induced vasculitis in human, Type 1 diabetes in human, Systemic lupus erythematosus in human
	Low-dose interleukin-2 [112]	Promote Treg cells recruitment	Increased Treg cells response	Alopecia areata in human
	Engineered IL-2 mutein [114, 115], IL-2/IL-2 mAb complex [116, 117]	Increase life-half of IL-2, selective expansion of Treg cells	Selectively activate and expand Treg cells	Mouse colitis model, cynomolgus monkey, type I diabetes, EAE, and xenogeneic graft-versus-host disease
	IL-4 [118], IL-12 [121]	Delay donor allograft rejection /Increased Treg cells survival and granzyme expression in Treg cells	Treg cells survival and function promotion	GVHD transplantation Model/−
	IL-5 [119]	Induce Ag-specific tolerance	Increased antigen-specific Treg cells	Experimental autoimmune neuritis
	IL-7 [120]	Maintain memory Treg cells in the steady state	mTreg cells maintenance	T cell receptor-alpha-deficient mice
	IL-15 [122]	Impact the balance of Treg cells and Th17 cells	Successful suppression of Treg cells	Inflammatory bowel disease mouse model
	IFN-γ [123]	Conversion of CD4^+^CD25^−^ T cells to CD4^+^ Treg cells	Treg cells induction	Experimental autoimmune encephalomyelitis mouse model
	Anti-CD3 antibody [124, 125]	Selectively deplete pathological cells while expand Foxp3^+^ Treg, Tr1, and Th3	Probably increased number and function	Multiple autoimmune models
In vitro	Adaptive transfer of Collagen-specific Treg [126, 127, 171]	Damping the proliferation of effector T cells, More Treg cells migrate into LN near the injection joint	Increased antigen-specific Treg number	Collagen-induced arthritis mouse model/ collagen antibody-induced rat arthritis model
	exogenous regulatory T cells transfusion [128]	increased proportion of endogenous Treg cells, RASF apoptosis, reduced B cells	Increased exogenous number Treg cells	Collagen-induced arthritis mouse model, RA synovial fibroblast cells
	Transfer of Ag-specific PSC-Tregs [134]	Suppress the development of IL-17 producing cells in an Ag-dependent fashion	Increased antigen-specific iPSC-Treg cells	Ag-induced arthritis animal model
	Transfer of induced Treg cells derived from naïve CD4^+^ T cells [135]	Suppress the activation of T cells	Increased absolute number of Treg cells	Chronic colitis mouse model
	Transfer of ex vivo expanded Treg cells [136–138]	Inhibit effector T cells, inhibition of T cells, B cells, as well as osteoclast-mediated bone destruction	Increased proportion of circulating Treg cells	Patients with acute graft-versus-host disease, CIA mouse model

### In vivo* strategies*

Immunomodulatory drugs targeting Treg cells in vivo represent a possible strategy to improve Treg cell functions and to maintain immune tolerance in RA. This approach includes the application of cytokines as well as the treatment with specific antibodies.

#### Preclinical studies using animal models

A series of preclinical studies revealed that it is possible to increase absolute Treg cell numbers with cytokines or antibodies and to improve Treg cell functions. For example, oral 1,25-dihydroxyvitamin D3 was reported to be able to stabilize Treg cells by enhancing TGF-beta and Foxp3 gene expression, thereby increasing absolute Treg cell numbers and reducing IL-6 expression in mice with lupus-like disease [106]. In addition, short-chain fatty acids (SCFAs) contribute to Treg cell expansion in vitro and in vivo [107]. Moreover, progesterone promotes the suppressive activity of Treg cells and increases the stability of Treg cells in inflammatory tissues through the mTOR pathway [108]. Furthermore, vitamin C was reported to be necessary for Foxp3 expression [109]. All these drugs prove that the suppressive capacity of Treg cells can be modulated in vivo.

#### In vivo* strategies targeting Treg cells*

IL-2 receptor alpha chain (CD25) is highly expressed on Foxp3 Treg cells compared to effecter CD4^+^ T cells. Enhancing IL-2 signaling in Treg cells improves the immunosuppression mediated by Treg cells. However, IL-2 signaling is also necessary for the activation and proliferation of proinflammatory T cells. Therefore, some research groups developed immunomodulatory drugs which can prevent the activation of effector T cells by IL-2. Low-dose IL-2 leads to expansion of Treg cell populations and recovery of Treg cell suppression in vivo [110–113]. Engineered IL-2 has the ability to selectively activate and expand regulatory T cells [114, 115]. Furthermore, IL-2/IL-2 mAb complex also showed potent selective immune regulation on T cells in vivo probably by conformational changes, although the mechanism of this effect remains unclear [116, 117]. In addition, other cytokine-based strategies have the potential to improve Treg cell functions. These cytokines include IL-4, IL-5, IL-7, IL-12, IL-15, and IFN-γ [118–123]. Although these strategies have been tested in mouse models of organ transplantation, type 1 diabetes and other autoimmune disorders, these cytokines have not been used to ameliorate experimental autoimmune arthritis or RA, so far. However, promising results observed in other autoimmune diseases justify further research on the effect of immunosuppressive cytokines on arthritis. Interestingly, selective depletion of effector T cells by anti-CD3 antibodies helps to improve immune tolerance in autoimmune patients and mouse models by preferentially depleting pathogenic cells while preserving Treg cells [124, 125].

### In vitro* strategies*

#### Preclinical studies involving mouse models

Asnagli et *al.* reported that collagen-induced Treg cells was effective in the treatment of experimental autoimmune arthritis [126]. The efficiency of Treg cell transfer in rats with CIA varies dependent on the stage of disease. In the early stages, Treg cell transfer reduces the symptoms while it shows no effect in later stages because the ability of Treg cells to migrate to lymph nodes is impaired [127]. A recent report has shown that transfusion of exogenous Treg cells in CIA mice can significantly improve the severity of arthritis by increasing the proportion of Treg cells in the spleen and in the peripheral blood [128]. Several phase I/II clinical trials with Treg cells have been performed, so far [101, 129, 130]. Th17 cells are a highly proinflammatory T cell subset in autoimmune arthritis [24, 131–133], Haque et al. have shown that the transfer of functional antigen-specific Treg cells, which were obtained by reprogramming induced pluripotent stem cells, ameliorated the development of CIA by suppressing pro-inflammatory Th17 cells [134]. Ex vivo generated iTreg cells from purified mouse CD4 + T splenocytes showed equivalent suppressive activity as freshly isolated nTreg cells in a mouse model of chronic colitis [135]. Moreover, adoptive cell therapy (ACT) with Treg cells from the peripheral blood, which are expanded in vitro by stimulation with anti-CD3 and anti-CD28 antibodies in the presence of IL-2, has shown promising results. ACT can prevent the development of CIA [136] by inhibiting T cells, B cells and osteoclast-mediated bone destruction [137, 138].

#### Clinical trials with Treg cells in RA patients

Several preclinical studies in arthritis mouse models have shown the effectiveness and safety of adoptive Treg cells and their potential to ameliorate clinical symptoms. Clinical trials have shown that adoptive Treg cell transfer was safe and achievable in kidney transplant recipients (NCT02091232) [104] and was associated with a reduced rate of infectious complications in type 1 diabetes (NCT01210664) [139]. There are some ongoing clinical trials involving patients with other types of diseases, including acute graft-versus-host disease (NCT01795573), steroid-refractory chronic graft-versus-host-disease (NCT03683498), and pemphigus (NCT03239470). These clinical trials may provide useful information for the development of Treg cells-based clinical trials in RA. So far, adoptive Treg cell transfer has not been used in clinical trials involving RA patients.

### Limitations, challenges and opportunities for Treg-based therapies

Despite recent advances, Treg cell-based therapies remain challenging. First, the number of nTreg cells is low in the peripheral blood and the required number of cells can hardly be obtained without ex vivo expansion of the Treg cell population. Second, iTreg cells are not as stable as nTreg cells. This means that iTreg cells will lose their suppressive function by reducing Foxp3 expression in the absence of stimulating cytokines [140]. However, several pieces of evidence show that it is possible to expand Treg cells which have effective suppressive functions in vitro based on good manufacturing practice-compliant (GMP-compliant) protocols [141–148]. These GMP-compliant protocols can be used for adoptive Treg cell transfer. In addition, Treg cells should be specific enough to target cells. Chimeric antigen receptors can help to increase the specificity, thereby re-directing Treg cells against immunogenic antigens and restore immune tolerance.

#### Limited number of available Treg cells in the peripheral blood

Lanni et *al.* reported that freshly isolated Treg cells can be used for clinical applications [149], but the low frequency of Treg cells in the peripheral blood restricts their use in clinical trials. It has been shown that conventional T cells can convert into Treg cells in mice by forced expression of Foxp3. In humans, the situation seems to be by far more complicated as it is not possible to generate potent suppressive human Treg cells in vitro by retroviral gene transfer mediated overexpression of Foxp3 [150–153]. However, Allan et *al.* reported the successful use of lentivirus for the transduction of FoxP3, thereby efficiently converting effector T cells into Treg cells [154]. However, further confirmation is required and safety issues regarding viral gene transfer into human T cells have to be solved. Interestingly, Dijke et al. reported that discarded human thymuses from pediatric cardiac surgery can serve as a new efficient source of Treg cells. These Treg cells are characterized by enhanced survival, stable expression of FoxP3 and immunosuppressive functions [101] (See Table 1).

#### Instability of Treg cells

Transduction of Foxp3 into iTreg cells seems to be a promising approach to induce stable FoxP3 expression, to stabilize the Treg cell phenotype and to maintain the full suppressive ability of Treg cells [154]. Recently, Honaker et *al.* used homology-directed repair (HDR)-based gene editing to enforce stable and robust expression of endogenous Foxp3, showing suppressive activity of iTreg cells both in human and mice with inflammatory diseases [155]. The stability of iTreg cells might be important to develop the full potential of adoptive Treg transfer in future clinical trials. However, it remains unclear whether the efficiency of genetically engineered iTreg cells is comparable to freshly isolated ex vivo nTreg cells or peripheral Treg cells [156]. It has also been reported that the transcription factors Helios, Eos, IRF4, Satb1, Lef1, and GATA-1 are necessary to maintain the suppressive property of Treg cells [157–160]. These findings demonstrate the complexity of Treg cell functions and the challenges which have to be faced to obtain suppressive iTreg cells. It has been reported that epigenetic modifications are involved in the regulation of Treg cell stability [161], but the underlying mechanisms remain unclear.

#### Opportunities for Treg cells expansion

It has been reported that freshly isolated Treg cells can be expanded ex vivo under Treg skewing conditions [162], and expanded polyclonal suppressive human Treg cells can prevent graft rejection [163]. During the past several years, there are some pieces of evidence showing that it is feasible to expand a satisfying amount of Treg cells that keep their suppressive ability in vitro using GMP-compliant protocols (see Table 2). This approach seems promising for future clinical trials in this field. Some reports suggest different approaches, including retroviral gene transfer mediated overexpression of FoxP3 or engineered iTreg cells to increase the stability of their suppressive function. In most reports, Treg cells were expanded from natural Treg cells obtained from peripheral blood mononuclear cells whereas in one of these reports cryopreserved umbilical cord blood was used to obtain naïve Treg cells. Anti-CD3/CD28 beads, IL-2 and rapamycin or everolimus were used in these expansion conditions. It is worth highlighting that Landwehr et al. reported that they used an allogeneic B cell bank for the expansion of allospecific natural Treg cells, which showed a superior suppressive ability compared to polyclonal natural Treg cells [143]. Even though the expansion protocols vary among these reports, they all show that a satisfying amount of Treg cells can be obtained and that Treg cells keep their suppressive function. However, further investigation is required to determine how these Treg cells could contribute to the restoration of immune tolerance in clinical trials.

**Table 2:** Treg cells expanded with good manufacturing practice (GMP)-compatible protocols in human cells

Evidence	Cell origin	Mechanisms	Time to expand	Expansion conditions	Expansion effects	Suppressive ability	Possible application	Literature
1	Cryopreserved umbilical cord blood	Naïve Treg cells isolation and expansion in vitro	16 days	Artificial APC or CD3/CD28 beads, IL-2	Mean 2092-fold expansion to 1.26*10^9^	Effective suppression against responder T cells	Autologous adaptive cell transfer therapy	[141]
2	Peripheral blood	In vitro expansion	36 days	Anti-CD3/CD28 beads, rapamycin, IL2	300-fold expansion	Effective suppression function	Clinical trials	[142]
3	Peripheral blood	Expansion of natural Treg by allogeneic activated B cells	28 days	B cell lines, rapamycin, IL2	80- to 120-fold expansion	Superior suppressive ability compared to polyclonal natural Treg cells	Suppressing allogeneic skin graft rejection in vivo	[143]
4	Peripheral blood	Expansion Treg cells in vitro	21 days	Anti-CD3/CD28 beads, rapamycin, IL2	Not available	Retained its suppressive function for at least 1 year	Therapy for inflammatory and autoimmune disorders	[144]
5	Peripheral blood	mTOR inhibitor Everolimus based expansion	21 days	Anti-CD3/CD28 beads, everolimus, IL2	Around 100-gold expansion	Suppression comparable with those induced with rapamycin	Clinical application in transplantation	[146]
6	Peripheral blood	Expansion of Treg cells in vitro	28 days	Anti-CD3/CD28 beads, rapamycin, IL2	25- to 200-fold increase	Suppressive function restored by expansion	Adoptive therapy based on Treg cells	[147]
7	Peripheral blood	In vitro expansion	21 days	Anti-CD3/CD28 beads, IL2	107- to 196-fold expansion	Effective suppression against effector cells	Clinical trials for translational research	[148]
8	Peripheral blood	Expansion in vitro	19 days	Anti-CD3/CD28 beads, IL2	70- to 185-fold expansion	Effective suppression	For mRNA-engineered Treg for further clinical application	[145]

#### Antigen-specificity of Treg cells and use of chimeric antigen receptors

Treg cells express a spectrum of molecules that helps them to transmigrate into inflammatory sites, including CCR6, CXCR4 and CXCR5 [164]. Evidence obtained from animal studies shows that antigen-specific Treg cells are more effective than polyclonal Tregs [155, 165–171]. A study about Treg cell transfer in a mouse model of myocardial infarction-induced ventricular remodeling has demonstrated that transferred Treg cells predominantly accumulate in the spleen rather than in inflammatory tissues [172]. Therefore, antigen-specific Treg cells are believed to be more suitable for clinical applications. Recently, we reported the role of post-translational modification of VASP for reduced migration of Treg cells in RA [24]. VASP might serve as a new potential specific target for engineered Treg cells. High VASP expression could increase the migration of Treg cells into inflamed synovial tissues in RA patients. To date, several strategies have been developed to obtain antigen-specific Treg cells: T cell receptor overexpression, antigen-stimulated expansion and chimeric antigen receptors (CAR). However, the first two methods are limited by low cell numbers, complex manufacture procedures and patient-specific TCRs. In contrast, CARs seem to be a promising tool for the generation of antigen-specific Treg cells. Various CARs have been used for the experimental treatment of autoimmune diseases. For example, CD19-CAR transfer was reported to be effective to suppress pathogenic B cells in autoantibody-mediated autoimmune disease. CD19-CAR Treg cells derived from naïve CD4^+^CD25^high^CD127^low^CD45RA^+^ cells maintained their suppressive functions, including high expression levels of TGF-beta and Foxp3 as compared to CD19-CAR Treg cells derived from CD4^+^CD25^high^CD127^low^CD45RO^+^ memory cells [173].

Recently, CAR-T cells targeting specific citrullinated peptide epitopes, including citrullinated vimentin (cVIM), citrullinated type II collagen (cCOII), citrullinated fibrinogen (cFib), citrullinated tenascin-C (cTNC-5), and cyclocitrulline peptide (CCP-1) were tested in vitro using T cells from CIA mice or RA patients. These CAR-T cells not only specifically recognized and killed anti-COII antibody-secreting B cells from CIA mice but also diminished specific autoreactive B cells in RA patients [174]. Noteworthy, there are individual autoantigen expression profiles in RA patients, making it difficult to develop specific CAR-T cells for a larger cohort of patients. However, it is possible to develop customized CAR-T cells for patients with severe diseases and organ manifestations. Moreover, Whittington et al. reported recently that DR1-CII CAR-T cells can specifically and effectively recognize and kill CD4^+^ T cells which are specific for the CII autoantigen in vivo in CIA mice. This leads to reduced autoantibody production, decreased collagen II-specific CD4^+^ T cell response and diminished severity of autoimmune arthritis in mice [175]. Furthermore, HLA-A*02 CAR-Treg cells were generated and have shown to be highly effective in preventing immune responses mediated by HLA-A*02 in both, human and mice [176]. Currently, there is no clear evidence showing that CAR-Treg cells could be used to cure RA, even though the aforementioned specific CAR-T cells seem to be very promising. Therefore, further investigations are need, especially to test the efficacy of CAR-Treg cells in vivo in RA patients and in arthritis mouse models.

In addition, co-stimulation with CD28/CTLA-4/B7 domains plays an important role in the homeostasis and function of Treg cells [177, 178]. The intracellular domain of CD28 is often integrated in CARs along with the intracellular domain of CD3. Dawson et *al.* reported that co-receptor signaling domains improve the efficacy of CAR-Treg cells [179]. The authors compared ten different co-receptor signaling domain-CARs and analyzed the gene expression profiles and the functions of human Treg cells. They demonstrated that a CAR encoding CD28 is superior to CARs with other intracellular signaling domains, including ICOS, CTLA-4, PD-1, OX40, GITR, 4-1BB, and TNFR2 [179]. Interestingly, 4-1BB and TNFR2 CARs are less effective regulator of the methylation status of TSDR within Foxp3, thereby inducing Treg cells with reduced stability. Furthermore, Boroughs et al. have reported that CD28-based CAR-Treg cells maintain their suppressive function in contrast to 4-1BB-CARs [180]. Due to the enhanced specificity of antigen-specific Treg cells, lower numbers of engineered iTreg cells are needed for adaptive Treg cell transfer as compared to nTreg cells.

The half-life time of CAR-T cells was reported to be relatively short after passive transfer into CIA mice and it was not possible to detect CAR-T cells in mice later than day 10 after adoptive cell transfer [175]. However, CAR-CD8 + Treg cells are still functional and can activate T cells in vivo for at least 80 days [181]. Moreover, CAR-CD4 + Treg cells can still be recruited at least 40 days after transfer into mice with skin grafts [170]. The mechanisms regulating the survival time of CAR-T cells and CAR-Treg cells is not clear yet, but it seems that the half-life time of CAR-Treg cells is superior to the half-life time of CAR-T cells. Further efforts will help to improve antigen-specific Treg transfer and to use CAR Treg cells for clinical application.

## Conclusion

Treg cell transfer and maintenance of Treg cell functions is a promising strategy to improve the treatment of RA. Preclinical and clinical trials have demonstrated the efficiency of Treg cell transfer. CAR-Treg cells may be a helpful tool to treat or even cure autoimmune diseases. However, a better understanding of Treg cells and their regulatory mechanisms is still required to improve the clinical application of Treg therapy in RA. The best time point for Treg cell therapies and the optimal dosage of Treg cells or Treg cell promoting drugs need to be determined. Taken together, Treg cell therapies have the potential to revolutionize RA therapy but intensive research is still needed to evaluate and improve this therapeutic approach.

## Data Availability

Not applicable.
